# Supplementary material for: QTc Interval Prolongation as an Adverse Event of Azole Antifungal Drugs: Case Report and Literature Review
Source: Microorganisms. 2024 Aug 8;12(8):1619. doi: 10.3390/microorganisms12081619 (PMC11356777; doi:10.3390/microorganisms12081619)
Supplement: Supplementary file 1 [file microorganisms-12-01619-s001.zip › microorganisms-3136897-supplementary.pdf]

Case	First author, year, reference	Age (years), sex	Underlying disease	Pre-existing cardiovascular disease	Organism	Fungal infection focus	Number of types of antifungal drugs	Antifungal drug regimen	Antifungal drug dosage	Antifungal drug administration purpose	Other drugs potentially causing QTc prolongation
1	Wassmann, 1999 [19]	59, F	Liver cirrhosis and peritonitis	None	*Candida albicans*	Peritonitis	Single	Fluconazole	400–800 mg/day for 5 weeks	Therapeutic	None
2	Dorsey, 2000 [22]	57, F	Mild diabetes mellitus, chronic depression, remote deep venous thrombosis with pulmonary embolism, and chronic renal insufficiency	Hypertension	*Cryptococcus* spp.	Pneumonia	Single	Fluconazole	Unknown	Therapeutic	Amitriptyline (List 3) and sertraline (List 3)
3	Hinterseer, 2006 [23]	25, F	Acute respiratory distress syndrome, septic shock, and multiple organ failure	Dilated left ventricle with severely impaired left ventricular function	Not detected	—	Single	Fluconazole (empirical administration)	400 mg every 24 h	Prophylactic	Erythromycin (List 1) and norepinephrine (List 4)
4	Pham, 2006 [24]	33, F	Systemic lupus erythematosus, hemolytic anemia, pulmonary eosinophilia, constrictive pericarditis, and proliferative glomerulonephritis	Hypertension	*Candida albicans*	Pneumonia	Single	Fluconazole	200 mg/day i.v. adjusted according to renal function	Therapeutic	Albuterol (List 4), citalopram (List 1), furosemide (List 3), isoproterenol (List 4), and pantoprazole (List 3)
5	Eiden, 2007 [25]	14, F	Acute myeloid leukemia and neutropenia	None	Unknown	Mucormycosis	Multiple (simultaneously and consecutively)	Liposomal amphotericin B to caspofungin, voriconazole to posaconazole	Unknown	Therapeutic	Piperacillin/tazobactam (List 3)
6	Esch, 2008 [26]	11, M	Neurofibromatosis-1, paraplegia, and neurogenic bowel/bladder dysfunction	None	*Candida albicans*	Septic shock	Single	Fluconazole	150 mg i.v. every 12 h	Therapeutic	Furosemide (List 3) and chloral hydrate (List 3)
7	Tacken, 2011 [27]	69, F	Tubo-ovarian abscess and pre-sacral abscess	Atrial fibrillation and hypertension	Not detected	—	Single	Fluconazole	Maximum of 100 mg	Prophylactic	Piperacillin/tazobactam (List 3), esomeprazole (List 3), propofol (List 1), and sevoflurane (List 1)

8	Aypar, 2011 [28]	15, M	—	Ventricular septal defect	*Trichosporon asahii*	Fungal endocarditis, intracranial hemorrhage, coma, and peripheral septic embolization to the left toe	Multiple (simultaneously)	Liposomal amphotericin B and voriconazole	3 mg/kg liposomal amphotericin B i.v. every 24 h; 6 mg/kg voriconazole i.v. every 12 h	Therapeutic	None
9	Aypar, 2011 [28]	12, F	Relapsed acute lymphoblastic leukemia	None	*Aspergillus* spp.	Pulmonary aspergillosis	Multiple (simultaneously)	Voriconazole and caspofungin	Unknown	Therapeutic	Imatinib mesylate (List 2)
10	Elbey, 2012 [29]	34, F	Nephrotic syndrome	Rheumatic mitral valve disease	Not detected	—	Multiple (consecutively)	Amphotericin B to voriconazole	200 mg voriconazole i.v. every 12 h	Prophylactic	Piperacillin/tazobactam (List 3)
11	Panos, 2016 [2]	26, F	Ectopic calcifications in the left hip joint, surgical site infection, and sepsis	None	MDR *Acinetobacter baumannii*	—	Multiple (consecutively)	Liposomal amphotericin B, voriconazole, and oral posaconazole (empirical administration)	Unknown	Prophylactic	None
12	Trang, 2017 [30]	22, F	Advanced cystic fibrosis	None	*Aspergillus fumigatus*, MDR *Mycobacterium abscessus*, and MDR *Pseudomonas aeruginosa*	Chronic lung infections	Single	Voriconazole	200 mg i.v. every 12 h	Therapeutic	Ciprofloxacin (List 1) and azithromycin (List 1)
13	Tilton, 2019 [31]	32, F	Mediastinal B-cell lymphoma and neutropenia	None	Not detected	—	Single	Fluconazole	Unknown	Prophylactic	Levofloxacin (List 1)
14	Yüksekgönül, 2021 [32]	16, F	Diffuse axonal injury and pneumonia	None	Not detected	—	Single	Fluconazole	Unknown	Prophylactic	None
15	Yuan, 2023 [33]	41, M	Acute myeloid leukemia	None	*Candida tropicalis*	Invasive fungal infection of the lung, liver, and blood	Multiple (simultaneously)	Micafungin and fluconazole	Unknown	Therapeutic	Dasatinib (List 2)
16	Present case, 2023	77, M	—	Chronic atrial fibrillation, heart failure, and old myocardial infarction	*Candida albicans*	Knee arthritis	Single	Fluconazole	200 mg p.o. every 12 h	Therapeutic	Amiodarone (List 1), esomeprazole (List 3), and trazodone (List 3)

Case	Serum electrolyte levels	Interval between antifungal drug administration and QTc prolongation detection	Longest QTc interval	TdP presence	Other cardiovascular events	Management of antifungal drug post-event	QTc interval change after episode occurrence	QTc prolongation and cardiovascular event treatment	Disease relapse	Outcome
1	Normal	Unknown	600 ms	Yes	None	Discontinuation	After 3 weeks, 423 ms	Cardiopulmonary resuscitation	No	Survived
2	3.1 mEq/L K	Unknown	Unknown	Yes	Syncope	Reduction	Unknown	Unknown	No	Survived
3	Normal K level; 0.5 mg/dL Mg	Unknown	510 ms	Yes	Ventricular fibrillation	Unknown	Unknown	External defibrillation, and magnesium sulphate and K i.v.	No	Survived
4	Normal	5 days	675 ms	Yes	None	Discontinued and then resumed after improvement of the QTc interval	Immediately after, 443 ms	Cardioversion, and Mg and isoproterenol i.v.	Yes (6 weeks later, once, after fluconazole re-administration)	Survived
5	Normal K level; 0.4 mg/dL Mg	5 days after voriconazole initiation	500 ms	Yes	Cardiac arrest	Discontinuation	After 1 month, 440–455 ms	Cardiac massage, magnesium sulfate i.v., nadolol p.o., and cardioverter–defibrillator implantation	Yes (3 months later, once)	Survived
6	3.1 mEq/L K; normal Mg level	5 days	490 ms	Yes	Pulseless monomorphic ventricular tachycardia	Discontinuation	After 4 months, 422 ms	Cardiopulmonary resuscitation and lidocaine i.v.	No	Survived
7	Normal	2 days	626 ms	Yes	None	Switch to caspofungin	QTc remained prolonged	Defibrillation and cardioverter–defibrillator implantation	No	Survived
8	Normal	During initial voriconazole administration	500 ms	Yes	None	Switch to caspofungin	Unknown	Defibrillation, and Mg and lidocaine i.v.	No	Survived

9	2.8 mEq/L K; 1.4 mg/dL Mg	11 days	570 ms	No	Ventricular bigeminy and trigeminy beats	Discontinued and then resumed after improvement of the QTc interval	After 12 h, 390 ms	Lidocaine, K, and Mg i.v.	No	Survived
10	Normal K level; 1.2 mg/dL Mg	4 days after voriconazole initiation	580 ms	Yes	None	Discontinued and then resumed after improvement of the QTc interval	After 2 days, 440 ms	Electrical cardioversion, emergent temporary pacemaker, and magnesium sulfate i.v.	Yes (several days later, once, after voliconazole re-administration)	Survived
11	Normal	11 days after voriconazole initiation	540 ms	Yes	Cardiac arrest	Discontinuation	Unknown	Cardiopulmonary resuscitation, defibrillation, and temporary pacemaker placement	No	Survived
12	Normal	QTc prolongation present prior to antifungal drug initiation	613 ms	No	None	Switch to isavuconazole	After 2 days, 468 ms; after 15 days, 442 ms; after 17 days, 470 ms	Isavuconazole i.v.	No	Survived
13	3.4 mEq/L K; normal Mg level	Unknown	551 ms	No	Syncope	Discontinuation	After 1 week, 443 ms	Unknown	No	Survived
14	Unknown	6 days	560 ms	Yes	None	Discontinuation	After 1 day, 540 ms; after 5 days, 402 ms	Lidocaine and propranolol i.v.	No	Survived
15	3.2 mEq/L K; 0.9 mg/dL Mg	25 days	520 ms	Yes	Ventricular fibrillation	Discontinuation	After 5 days, 402 ms	Cardiopulmonary resuscitation, electrical cardioversion, and isoproterenol, magnesium sulfate, and potassium magnesium aspartate i.v.	No	Survived
16	Normal	1 month	536 ms	Yes	None	Switch to micafungin	After 12 days, <450 ms	Implantable cardioverter–defibrillator shock, potassium chloride and magnesium sulfate i.v., and potassium L-aspartate p.o.	No	Survived

Supplementary Table S1. Characteristics of cases of QTc prolongation caused by azole antifungal drugs.
In this review, the assessment of the risk of QTc prolongation for each drug was conducted by referencing the QT drugs list from CredibleMeds [32]. Drugs were categorized into: List 1, drugs with a known risk for TdP; List 2, drugs with a possible risk for TdP; List 3, drugs with a conditional risk for TdP; and List 4, drugs to be avoided by patients with congenital long QT syndrome. Serum electrolyte levels indicate the values at the time of QTc prolongation events. K and Mg levels are considered normal if within the ranges of 3.5–5 mg/dL and 1.7–2.6 mg/dL, respectively. Abbreviations: F, female; i.v., intravenously; K, potassium; M, male; MDR, multidrug-resistant; Mg, magnesium; p.o., per os; TdP, torsade de pointes
